# Essential Metals and Trace Elements in Cereals and Their Derivatives Commercialized and Consumed in Cape Verde

**DOI:** 10.1007/s12011-022-03158-x

**Published:** 2022-02-28

**Authors:** Carmen Rubio-Armendáriz, Ángel J. Gutiérrez, Verena Gomes-Furtado, Dailos González-Weller, Consuelo Revert, Arturo Hardisson, Soraya Paz

**Affiliations:** 1 Department of Toxicology, Universidad de La Laguna, La Laguna, Tenerife, Canary Islands Spain; 2 Independent Health Regulatory Authority, Av. Cidade de Lisboa, Praia, Cape Verde; 3 Health Inspection and Laboratory Service, Canary Health Service, S/C de Tenerife, Tenerife, Canary Islands Spain; 4 Department of Physical Medicine and Pharmacology, University of La Laguna, Tenerife, Canary Islands Spain

**Keywords:** Cereals, Cereal derivatives, Essential elements, Trace elements, Dietary intake, Cape Verde

## Abstract

Cereals and their derivatives are basic foods in the human diet and a source of minerals, but the content of elements may vary depending on the type of cereal or its processing. The levels of Na, K, Ca, Mg, Fe, Cu, Zn, Mo, Co, and Mn have been determined in 126 samples of cereals and cereal derivatives (rice, corn *gofio*, corn flour, wheat flour, corn, and wheat) commercialized and consumed in Cape Verde using an inductively coupled plasma-optical emission spectrometer (ICP-OES) after a wet microwave digestion process. Some elements stand out in products such as corn gofio (K), wheat (Mg), and wheat flour (Fe). Negative correlations were found between Mo-Na and Na-Zn that could suggest interference between these elements. Bearing in mind the dietary intake evaluation and the guideline values provided by EFSA (European Food Safety Authority) or FAO (Food Authority Organization), the consumption of wheat (100 g/day) provides a notable contribution of Mo, Mn, Fe, and Mg. Considering the nutritional value of the analyzed essential and trace elements, the consumption of different cereals and their derivatives should be promoted.

## Introduction

Cereals have been a staple foodstuff in the human diet for more than 10,000 years. Nowadays, the most consumed cereals worldwide are wheat, rice, corn, barley, oats, and rye. These are used in the production of daily consumption products such as bread, cookies, pasta, and drinks. Global cereal production is now almost three billion tons according to data collected by the FAO (Food and Agricultural Organization) [1]. However, the nutritional value of cereals is affected by various factors such as soil quality, water composition, crop rotation, or the use of growth promoters. Therefore, the determination of the nutrient content in cereals, such as essential elements, is necessary to know their dietary intake.

The population of Cape Verde in 2020 was 555,988 inhabitants, and the nutritional status of the population has been previously evaluated and monitored [2–5]. According to data from the Ministry of Health and Social Security of the Government of Cape Verde, 11% of children under 5 years of age suffer from chronic malnutrition [3]. The latest data show that 37.7% of households in Cape Verde do not have economic access to safe, nutritious, and sufficient food [3] and 4.5% of children under 5 suffer from acute malnutrition [3]. At the national level, 52.7% of households have moderate to low diversity in their diet, but this includes the cereal group [2, 3] with a consumption of 84,820 tons in 2020. In Cape Verde, 84,000 of the 403,000 hectares of surface area are used for agriculture, and corn is one of the main crops grown on the islands [1]. In 2020, corn consumption was 30,157 tons (almost 150 g/day per citizen). Corn is the base ingredient of several traditional dishes in Cabo Verde, such as “*cachupa*, *cherém*, *couscous*, and *camoca*.” Rice is the second most consumed cereal (25,996 tons, almost 120 g/day per citizen) followed by wheat (16,907 tons, around 80 g/day per citizen) and wheat flour (11,760 tons, around 60 g/day per citizen) [6, 7].

Cereals and their derivatives are valued for their content of both major elements or macro elements (Na, K, Ca, Mg) and trace elements (Fe, Cu, Zn, Mo, Co, Mn). Macro elements (Ca, Mg, K, Na) are required by humans in high quantities for proper body functioning. Ca is an element in bone tissues. K and Na play a role in maintaining osmotic pressure and in the transmission of nervous impulses, and Mg is a cofactor in more than 300 enzymatic reactions. Trace elements (Fe, Cu, Co, Zn, Mn, Mo) are also needed for the proper functioning of the organism but are required in smaller quantities [8–13]. These trace elements are involved in numerous biological processes: Fe is involved in oxygen transport, Co is an element forming part of cobalamin or vitamin B12, and Mo, Mn, Zn, or Cu are components in many enzymes [14–16]. The Food and Agricultural Organization/World Health Organization (FAO/WHO) and the European Food Safety Authority (EFSA) have set recommended daily intake (RDI) values for the essential elements (Table 1).

**Table 1:** Guideline dietary intake values of essential elements for adults

Group	K (mg/day)	Na (mg/day)	Cu (mg/day)	Mo (mg/day)	Mn (mg/day)	Reference				
Women (≥ 18)	3500	2000	1.3	0.065	3	[13]				
Men (≥ 18)	3500	2000	1.6	0.065	3					
Group	Ca (mg/day)	Mg (mg/day)	Zn (mg/day)			Fe (mg/day)				Reference
			High bv	Moderate bv	Low bv	15% bv	12% bv	10% bv	5% bv	[17]
Premenopausal women (19–50 years of age)	1000	220	3.0	4.9	9.8	19.6	24.5	29.4	58.8	
Menopausal women (51–65 years of age)	1300	220	3.0	4.9	9.8	7.5	9.4	11.3	22.6	
Men (19–65 years of age)	1000	260	4.2	7.0	14.0	9.1	11.4	13.7	27.4	

*bv*., bioavailability

In the case of Cape Verde, with the aim of strengthening the national nutritional surveillance and of improving the nutritional framework, the authorities developed the National Food and Nutrition Plan setting out guidelines and strategies to be implemented in the 2021–2025 period, contemplating food programs, aligned with the new challenges and in accordance with international recommendations [3]. The National Food and Nutrition Plan includes cereals and their derivatives as they are one of the most accessible foods for the population of Cape Verde, and, therefore, it is important to evaluate their nutritional value. As an example, Cape Verdean legislation states that wheat flour, which is commercialized and available to the final consumer, should be fortified with Fe. According to the WHO (World Health Organization), flour fortification programs should include appropriate Quality Assurance and Quality Control (QA/QC) programs at mills as well as regulatory and public health monitoring of the nutrient content and assessment of the nutritional/health impacts of the fortification strategies [17, 18]. The concentration of iron in the final product depends on the nutrient extraction level and the type of compounds used in the fortification process (ethylenediaminetetraacetate-NaFeEDTA, ferrous sulfate, ferrous fumarate, ferrous) [17, 18].

Considering the importance of cereals and their derivatives in the diet, the aims of the present study are (i) to determine the content of essential elements (Na, K, Ca, Mg, Fe, Cu, Zn, Mo, Co, Mn) in cereals and their derivatives, (ii) to study the possible significative differences or correlations between the essential elements in these cereals/derivatives, and (iii) to assess the dietary intake of the analyzed elements from the consumption of these cereals/derivatives by the population.

## Materials and Methods

### Samples and Sample Treatment

#### Samples

A total of 126 samples of cereals and derivatives were acquired from the following two regions of Cape Verde: Santiago and São Vicente. Table 2 shows the analyzed samples, the location where the samples were collected, and the origin of the cereal if available on the label. The analyzed cereals were corn, wheat, and rice (white), and the cereal derivatives were corn flour, wheat flour (plain), and corn *gofio*. The analyzed samples of corn *gofio* contained sea salt and traces of wheat, soy, egg, milk, sesame, and almonds [19, 20]. Gofio is a product made from whole cereal grains (mainly, corn), including the husk, which are roasted and ground until a dark colored flour is obtained, and it may also contain salt as an additive [21].

**Table 2:** Analyzed cereal and derivatives samples according to origin and place of harvesting

Type	No. of samples	Sampling location	Product and market	Origin^a^
Rice (white)	56	Santiago	Bulk product, local market	Brazil, Vietnam, Thailand, Japan, the USA (California), Pakistan
Corn gofio^b^	6		Packed, local market	Unknown
Corn flour	10		Packed, local market	Portugal, the Netherlands
Wheat flour (plain)	17		Packed, local market	Portugal, France
Corn	13		Bulk product, local market	Argentina, France, Russia, South America
Wheat	2		Bulk product, local market	Russia, France
Rice (white)	10	São Vicente	Bulk product, local market	Brazil, Vietnam, Thailand, Japan, the USA (California), Pakistan
Corn gofio^b^	2		Packed, local market	Unknown
Corn flour	2		Packed, local market	Portugal, the Netherlands
Wheat flour (plain)	4		Packed, local market	Portugal, France
Corn	4		Bulk product, local market	Argentina, France, Russia, South America
Wheat	22		Bulk product, local market	Russia, France, Spain

^a^Main importers of Cabo Verde: CORIN—Comercio Geral S.A., Minimercado Matilde—Productos Alimentares, MOAVE—Moagem de Cabo Verde S.A., IMPORTEX—Comercio e Representaçoes, CIC—Companhia de Investimentos de Cereais de Cabo Verde, Bento S.A., ITOM—Distribuidora Limitada, Irmaos Correia LDA, Silos Marangatu—Segurança Alimentaria

^b^Contains sea salt and traces of wheat, soy, egg, milk, sesame, and almonds

#### Sample Treatment

One gram of each sample was weighed using laboratory scale balance (ME4002, Mettler Toledo, USA) in a Teflon tube (HVT50, Anton Paar, Austria). Four milliliters of nitric acid (HNO_3_) at 65% reagent quality (Sigma-Aldrich, Germany) and 2 mL of hydrogen peroxide (H_2_O_2_) (Sigma-Aldrich, Germany) were then added. Samples were subjected to a wet microwave digestion process (Multiwave Go, Anton Paar, Austria) (Table 3) [22]. The digestion process lasted for 1 h. Once the samples had been digested, they were placed in 10-mL volumetric flasks, made up to the mark with Milli-Q quality distilled water, and transferred to hermetic containers for measurement (in triplicate). The proportion was 1:10 (w/v).

**Table 3:** Instrumental microwave conditions for wet digestion of samples

No	Ramp (min)	Temperature (°C)	Time (min)
1	15′00″	50	5′00″
2	5′00″	60	4′00″
3	5′00″	70	3′00″
4	3′00″	90	2′00″
5	20′00″	180	10′00″

Microwave processing power, 850 W; limit temperature, 200 °C; cooling temperature, 50 °C

### Determination of the Content of Essential Elements

Inductively coupled plasma-optical emission spectrometry (ICP-OES) using the ICAP 6300 model (Duo Thermo Scientific, Waltham, MA, USA) with an attached auto sampler (Auto Sampler, CETAX model ASX-520) was used to determine the essential element contents [23]. Instrumental conditions, wavelengths, and limits of quantification are shown in Table 4.

**Table 4:** ICP-OES instrumental conditions, instrumental wavelengths, limits of quantification, and quality control of the method

ICP-OES instrumental conditions			
RF power	1150 W	Injection of the sample (flow pump)	50 rpm
Gas flow (nebulizer & auxiliary)	0.5 L/min	Stabilization time	0 s
Instrumental wavelengths (nm)		LOQ^a^ (mg/kg)	
Ca	317.9	19.95	
Co	228.6	0.06	
Cu	327.3	0.12	
Fe	259.9	0.09	
K	769.9	18.84	
Mg	279.1	19.43	
Mn	257.6	0.08	
Mo	202.0	0.02	
Na	589.6	36.55	
Zn	206.2	0.07	
Quality control method			
	Found conc. (mg/kg)	Certified conc. (mg/kg)	Recovery (%)
Ca^b^	1967 ± 113	1961.1 ± 158	99.7
Co^c^	0.09 ± 0.00	0.09 ± 0.00	100
Cu^d^	2.1 ± 0.2	2.09 ± 0.4	99.7
Fe^d^	14.1 ± 0.5	13.9 ± 0.3	98.9
K^b^	6970 ± 125	6858.5 ± 318	98.4
Mg^b^	580 ± 26.7	575 ± 25.7	98.1
Mn^d^	9.4 ± 0.9	9.3 ± 0.5	98.9
Mo^c^	0.09 ± 0.01	0.09 ± 0.02	99.4
Na^b^	8132 ± 942	8001.9 ± 476	98.4
Zn^d^	11.6 ± 0.4	11.4 ± 0.2	98.2

^a^LOQ (limits of quantification), calculated as 10 times the standard deviation (SD) resulting from the analysis of 15 targets under reproducibility conditions

^b^SRM 1548a typical diet

^c^SRM 1515 apple leaves

^d^SRM 1567a wheat flour

In determining the elements Na, Ca, K, and Mg, the certified standard IV-STOCK-2 (Inorganic Ventures Inc., Christiansburg, VA, USA) was used with a certified concentration of 0.01 mg/mL for each element indicated. The certified standard Multi-Element Std (SCP28AES, SCP Science, Quebec, Canada) with a certified concentration of 100 mg/L was used for the determination of Mn, Fe, Cu, Zn, Mo, and Co.

The validation parameters that were verified in this analytical method were specificity, precision (established as reproducibility), and accuracy (stated as recovery). These parameters were verified with the measurement, under reproducibility conditions, of above-mentioned reference materials (ten times each). The results of the verification procedure were the following: Specificity, the method was found to be free of spectral interference for each of the metals studied; Precision, this was confirmed for each of the metals, with a HORRAT_R_ value < 2; Accuracy, the recovery of the elements studied in the reference material was > 94% in all cases (Table 4), with no significant differences (*p* < 0.05). Therefore, the method used met the criteria of accuracy (established as recovery), precision (established as reproducibility), and specificity as established in the EC REGULATION No. 333/2007 [24] (Table 4).

### Statistical Analysis

GraphPad Prism 8.4.3 software for Windows™ was used for the statistical analysis, and the Anderson–Darling, D’Agostino and Pearson, Shapiro–Wilk, and Kolmogorov–Smirnov tests were applied [25, 26]. As data did not follow a normal distribution, the Mann–Whitney test was applied [27]. Values of *p* < 0.05 were considered statistically significant. The existence of possible significant differences between cereals and their derivatives (rice, corn gofio, corn flour, wheat flour, corn, and wheat) and between origins (Santiago and São Vicente) was studied, and a correlation study based on the Spearman correlation coefficient [28] was conducted to find possible positive and/or negative correlations between the different elements analyzed.

### Dietary Intake Assessment Calculations

The estimated daily intake (EDI) of the different essential and trace elements derived from the consumption of cereals and derivatives was calculated. The EDI is defined as the estimated amount of a substance and/or chemical element, in this case, an essential/trace element, which is ingested daily from a portion of food, in this case, the cereals and derivatives [29].

1 $$\mathrm{EDI }=\mathrm{ Cereal~consumption \times Element~concentration}$$

The different guideline values set for each one of the analyzed elements were used to evaluate the EDI (see Table 1). A contribution percentage to these guideline values was estimated for the daily consumption of each cereal/derivative and each essential element. Even though corn consumption in Cape Verde was 30,157 tons (almost 150 g/day per citizen), rice consumption 25,996 tons (almost 120 g/day per citizen), wheat 16,907 tons (around 80 g/day per citizen), and wheat flour 11,760 tons (around 60 g/day per citizen), a 100 g/day portion of every cereal or cereal derivative was considered as the daily consumption [6].

2 $$\mathrm{Contribution }(\mathrm{\%}) = \frac{EDI}{Reference~intake~value}\times100$$

## Results and Discussion

### Content of Essential Elements in Cereals and Derivatives

Wheat samples had the highest mean average level of Mg (806 ± 358 mg/kg ww), and the contents of Mn (18.5 ± 13.6 mg/kg ww) and Zn (15.3 ± 4.70 mg/kg ww) in wheat samples are also noteworthy (Table 5). This fact is important because a deficient intake of Mg is related to cardiovascular diseases, type 2 diabetes, etc. [30]. Studies carried out by Laskowski et al. [31] showed that cereals are the main source of Mg in the diet. Wheat flour had the highest mean average concentration of Na (294 ± 1254 mg/kg ww) and Ca (191 ± 68.6 mg/kg ww), but wheat flour was found to have a lower Mg concentration (416 ± 274 mg/kg ww). These data suggest that the processing of cereals can decrease their nutritional quality [30].

**Table 5:** Mean average concentrations (mg/kg) and standard deviations of the analyzed elements in the samples from Cape Verde

Element	Rice		Corn		Corn flour		Wheat flour		Corn gofio		Wheat	
	Mean ± SD	Min–max	Mean ± SD	Min–max	Mean ± SD	Min–max	Mean ± SD	Min–max	Mean ± SD	Min–max	Mean ± SD	Min–max
Ca	65.4 ± 73.3	0.00 − 349	88.6 ± 93.0	0.00 − 308	58.0 ± 96.7	0.00 − 297	191 ± 68.6	37.6 − 272	99.7 ± 94.3	25.3 − 266	185 ± 115	29.9 − 298
K	873 ± 828	43.5 − 3540	2068 ± 793	572 − 3060	1295 ± 772	39.2 − 2690	1783 ± 715	801 − 3310	2503 ± 697	1590 − 3270	2371 ± 1221	0.00 − 3490
Na	< LOQ				42.9 ± 142	0.00 − 472	294 ± 1254	0.00 − 5470	< LOQ			
Mg	251 ± 257	0.00 − 1280	580 ± 303	105 − 923	297 ± 252	0.00 − 810	416 ± 274	196 − 1030	734 ± 231	446 − 1080	806 ± 358	106 − 1200
Co	< LOQ											
Cu	1.78 ± 1.99	0.00 − 14.4	1.16 ± 0.60	0.60 − 3.15	0.70 ± 0.33	0.15 − 1.30	1.44 ± 0.42	0.82 − 2.42	1.37 ± 0.52	0.69 − 2.11	2.96 ± 2.98	0.94 − 12.7
Fe	4.54 ± 8.93	0.13 − 54.4	12.8 ± 6.91	1.34 − 28.6	11.5 ± 17.5	0.80 − 59.1	36.8 ± 27.1	2.33 − 83.4	32.5 ± 14.4	10.5 − 52.9	24.1 ± 17.4	1.45 − 71.2
Mn	7.15 ± 4.93	0.00 − 32.8	4.97 ± 5.40	0.96 − 23.9	2.55 ± 2.49	0.00 − 7.26	8.42 ± 6.50	3.26 − 28.3	8.06 ± 8.57	0.62 − 22.5	18.5 ± 13.6	3.11 − 41.6
Mo	0.52 ± 0.24	0.00 − 1.34	0.43 ± 0.30	0.09 − 1.28	0.17 ± 0.14	0.02 − 0.44	0.40 ± 0.31	0.15 − 1.34	0.55 ± 0.30	0.18 − 1.05	0.82 ± 0.41	0.35 − 1.64
Zn	12.5 ± 11.2	0.24 − 95.2	9.99 ± 3.91	4.05 − 15.4	5.58 ± 3.59	0.27 − 12.1	8.84 ± 4.03	4.50 − 17.6	13.4 ± 4.33	7.57 − 19.2	15.3 ± 4.70	10.6 − 22.9

LOQ (limits of quantification): Na (36.55 mg/kg), Co (0.06 mg/kg)

*SD* standard deviation

Corn *gofio* had the highest mean average content of K (2503 ± 697 mg/kg ww). As mentioned above, the concentrations of certain elements may be higher in “*gofio*” because this derivative is made with wholegrain cereal including the husk [32, 33].

**Fig. 1:**
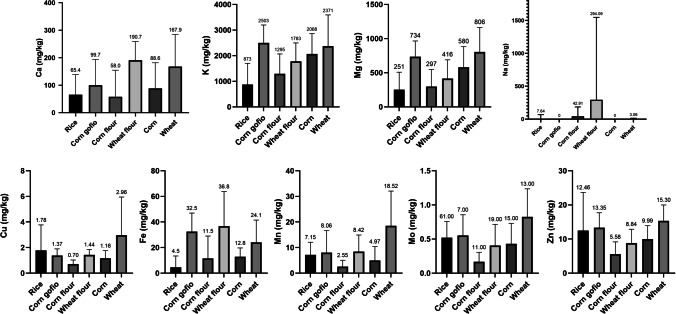
Box graph of the statistical analysis by cereal type

Figure 1 shows the comparison in the content of the elements for each sample group analyzed. Due to the disparity between the samples analyzed, the standard deviations (SD) are sometimes higher than the mean concentrations. Not only did the commercialized samples in Cape Verde have different origins (sometimes unknown) but some samples contained added fortified ingredients.

TatahMentan et al. [34] determined essential elements in white rice from the USA, Thailand, India, and Italy and found lower levels of Mg (260 mg/kg), Ca (57 mg/kg), K (833 mg/kg), and Fe (4.3 mg/kg) than the results of the present study, whereas the mean average concentration of Mn (11 mg/kg) was higher in white rice from the USA and Thailand, India, and Italy. Zn concentrations in white rice (12.5 mg/kg) from the research did not have significant differences with the commercial rice in Cape Verde (12.5 mg/kg).

Other authors have also reported that the concentrations of the analyzed elements in the cereals and derivatives commercialized in Cape Verde differ from both the EFSA food composition database and the INSA (Institute for Research on Nutrition and Food Safety) food composition table [35, 36].

An example of the above is the case of Ca; Ca concentrations in the different types of cereals analyzed in Cape Verde in the present study were lower than the mean average Ca values reported both by EFSA (wheat flour, 351.7 mg/kg; common wheat grain, 285 mg/kg; polished rice, 102 mg/kg; corn flour, 113.3 mg/kg) and INSA (wheat flour, 280 mg/kg; raw dry corn, 140 mg/kg; corn flour, 80 mg/kg) (Table 6). Furthermore, the K content was lower in the analyzed samples than the mean average K concentration reported in the INSA and EFSA databases (common wheat grain, 3171.4 mg/kg; polished rice, 1187 mg/kg; corn flour, 1667 mg/kg; corn grain, 2900 mg/kg) [35, 36]. In the case of Mg, the recorded concentrations were higher in the samples of wheat and corn flour commercialized in Cape Verde than the values reported by EFSA (wheat flour, 183.3 mg/kg) and INSA (corn grain, 80 mg/kg) [35, 36].

**Table 6:** Statistical differences between the analyzed samples and *p* value

Element	Rice	Corn	Corn flour	Wheat flour	Corn gofio	Wheat
Ca	vs corn flour (0.0027) vs wheat (0.011)	vs corn flour (0.0408) vs wheat flour (0.0015)	vs wheat flour (0.0008)	vs rice (< 0.0001)	vs corn flour (0.0317)	vs corn flour (0.003)
K	vs corn flour (0.015) vs wheat flour (< 0.0001)	vs corn flour (0.0246) vs rice (< 0.0001)	vs corn gofio (0.0041) vs wheat (0.0246)	vs corn flour (0.0452)	vs wheat flour (0.0223) vs rice (< 0.0001)	vs rice (0.0056)
Na	Non detected	Non detected	Non detected	Non detected	Non detected	Non detected
Mg	vs corn gofio (< 0.0001) vs wheat (< 0.0001)	vs corn flour (0.0204) vs rice (0.0001)	vs corn gofio (0.0044) vs wheat flour (0.0161)	vs rice (0.0003)	vs wheat flour (0.0056)	vs corn flour (0.0069) vs wheat flour (0.0096)
Co	Non detected	Non detected	Non detected	Non detected	Non detected	Non detected
Cu	vs corn flour (< 0.0001)	Non detected	vs rice (< 0.0001)	Non detected	Non detected	vs rice (0.0003)
Fe	vs corn flour (0.0036) vs wheat flour (< 0.0001)	vs wheat (0.0176) vs rice (< 0.0001)	vs corn gofio (0.0083) vs wheat flour (0.0017)	vs corn (0.0124)	vs corn (0.0029) vs rice (< 0.0001)	vs rice (< 0.0001)
Mn	vs wheat (0.0126)	vs rice (0.0001) vs wheat (0.0008)	vs rice (< 0.0001)	vs corn flour (0.0002) vs corn (0.0004)	vs wheat (0.0456)	vs corn flour (0.0002)
Mo	vs corn flour (< 0.0001) vs wheat flour (0.0136)	vs corn flour (0.0015)	vs wheat flour (0.0005)	vs wheat (0.0008)	vs corn flour (0.002)	vs rice (0.0085) vs corn flour (< 0.0001)
Zn	vs corn flour (< 0.0001)	vs corn flour (0.0056) vs wheat (0.0201)	vs corn gofio (0.0028) vs wheat (< 0.0001)	vs rice (0.0056) vs corn flour (0.0120)	vs wheat flour (0.0121)	vs wheat (0.0016) vs wheat flour (0.001)

The differences in levels confirm that the origin of the crop/cereal and the derivatives’ manufacturing process may impact the metal content of final products. This is the reason why the authors of the present work believe that any dietary exposure assessment should be carried out considering updated data obtained from each population under study.

Significant differences (*p* < 0.05) were found between all types of cereals and derivatives (rice, wheat, corn, corn gofio, wheat flour, corn flour) for almost all the elements determined, except for Na and Co. These differences may be associated with various factors. Not only do the intrinsic characteristics of the plant and the soil in which the cereal is grown (pH, organic matter, carbonate, and oxide content, etc.) directly influence the content of elements, but there are also other important factors such as the climatic conditions of the region where these crops are grown [37]. A recent study carried out by Soares et al. [38] reported a noteworthy interaction between individual climatic factors and the content of nitrogen (N), potassium (K), iron (Fe), and phosphorus (P) in cultivation soils.

The results of the present research should be viewed considering some limitations. Not knowing the origin of some of the analyzed samples may have a significant influence in the final element content of the cereal and derivatives, and therefore, this is considered a limitation in the results and discussion of this study. The importance of informative labeling that indicates the country and area of origin of the cereal is again pointed out as an essential component of an exposure assessment. Therefore, the authors suggest that every product sold to the public should provide information regarding its cultivation area (origin) on the label. Some of the secondary ingredients may enrich the final product in terms of the analyzed elements here, and as such the final concentration observed may not be completely attributable to the main cereal ingredient. Nevertheless, this limitation does not affect the exposure assessment estimation because the portion of the final product consumed by the Cape Verde population is what matters here.

### Study of Correlations Between Elements

**Fig. 2:**
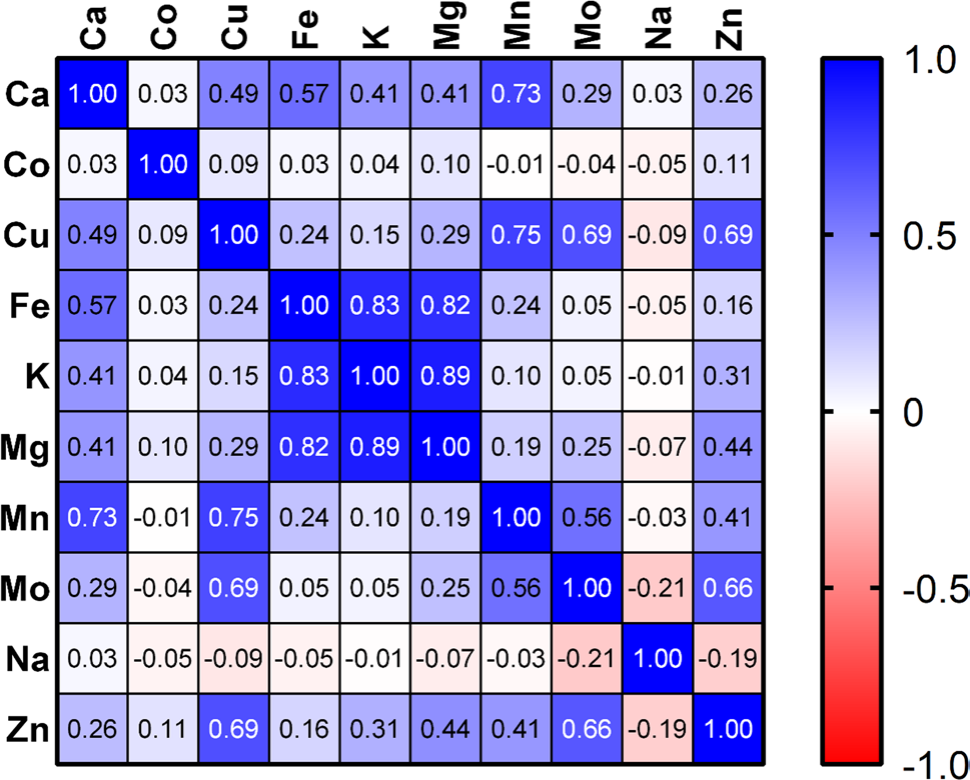
Correlations Spearman coefficient results

Figure 2 shows the results of the Spearman *r* correlation study. Positive correlations were found between Mn-Ca, Fe-Ca, Mn-Cu, Mo-Cu, Mo-Cu, Zn-Cu, Cu-Ca, K-Fe, Mg-Fe, Ca-Fe, K-Mg, Mn-Mo, and Mo-Zn. Positive correlations indicate the absence of competitiveness between elements and the simultaneous presence of elements without interference. The literature on the correlations between essential and trace elements in cereals is scarce. Studies conducted by Ŝimic et al. [39] determined positive correlations between Cu-Fe and Fe-Mo in corn. Although the physiological mechanisms between elements are not clearly known, the above authors suggest that the positive correlations found imply the possibility of simultaneously improving the element content in the corn grain [39]. Negative correlations were found between Mo-Na and Na-Zn and suggest interference between these elements. These data should be considered with caution because, in some of the cereal products analyzed, the manufacturer has added salt, which interferes with the study of correlations. Without any doubt, it is necessary to carry out more studies on correlations between essential and trace elements in cereals and their derivatives.

### Dietary Intake Evaluation

Tables 7, 8, and 9 show the estimated daily intake (EDI) of the analyzed elements assuming a consumption of 100 g/day of the analyzed cereals or derivatives. The evaluation of the dietary intakes was performed considering the “dietary reference values (DRV)” set by FAO [17] and EFSA [13].

**Table 7:** Estimated daily intake (EDI) of the essential elements according to cereal type and derivatives for a consumption of 100 g/day

Element	EDI (mg/day)					
	Rice	Corn	Corn flour	Wheat flour	Corn gofio	Wheat
Ca	6.54	8.86	5.80	19.1	9.97	18.5
K	87.3	207	130	178	250	237
Na	-	-	4.29	29.4	-	-
Mg	25.1	58.0	29.7	41.6	73.4	80.6
Fe	0.45	1.28	1.15	3.68	3.25	2.41
Cu	0.18	0.12	0.07	0.14	0.14	0.30
Co	-	-	-	-	-	-
Mo	0.05	0.04	0.02	0.04	0.06	0.08
Mn	0.72	0.50	0.26	0.84	0.81	1.85
Zn	1.25	0.99	0.56	0.88	1.34	1.53

**Table 8:** Contribution percentages to the guideline values for men (19–50 years of age), women (premenopausal, 19–50 years of age), and women (postmenopausal (19–65 years of age) set by the FAO [17]

Element	Group	%Contribution to the guideline values					
		Rice	Corn	Corn flour	Wheat flour	Corn gofio	Wheat
Ca	Men	0.65	0.89	0.58	1.91	1.00	1.85
	Women (premenopausal)	0.65	0.89	0.58	1.91	1.00	1.85
	Women (postmenopausal)	0.50	0.68	0.45	1.47	0.77	1.42
Mg	Men	9.65	22.3	11.4	9.65	16.0	36.6
	Women (pre- and postmenopausal)	11.4	26.4	13.5	11.4	18.9	31.0
Zn^a^	Men	29.8	23.6	13.3	21.0	31.9	36.4
	Women (pre- and postmenopausal)	41.7	33.0	18.7	29.3	44.7	51.0
Zn^b^	Men	17.9	14.1	8.0	12.6	19.1	21.9
	Women (pre- and postmenopausal)	25.5	20.2	11.4	18.0	27.3	31.2
Zn^c^	Men	8.9	7.1	4.0	6.3	9.6	10.9
	Women (pre- and postmenopausal)	12.8	10.1	5.7	9.0	13.7	15.6
Fe^d^	Men	4.95	14.1	12.6	40.4	35.7	26.5
	Women (premenopausal)	2.30	6.5	5.9	18.8	16.6	12.3
	Women (postmenopausal)	6.00	17.1	15.3	49.1	43.3	32.1
Fe^e^	Men	3.95	11.2	10.1	32.3	28.5	21.1
	Women (premenopausal)	1.84	5.2	4.7	15.0	13.3	9.8
	Women (postmenopausal)	4.79	13.6	12.2	39.1	34.6	25.6
Fe^f^	Men	3.28	9.3	8.4	26.9	23.7	17.6
	Women (premenopausal)	1.53	4.4	3.9	12.5	11.1	8.2
	Women (postmenopausal)	3.98	11.3	10.2	32.6	28.8	17.6
Fe^g^	Men	1.64	4.7	4.2	13.4	11.9	8.8
	Women (premenopausal)	0.77	2.2	2.0	6.3	5.5	4.1
	Women (postmenopausal)	1.99	5.7	5.1	16.3	14.4	10.7

^a^High bioavailability

^b^Moderate bioavailability

^c^Low bioavailability

^d^15% bioavailability

^e^12% bioavailability

^f^10% bioavailability

^g^5% bioavailability

**Table 9:** Contribution percentages to the guideline values for men (≥ 18) and women (≥ 18) set by EFSA [13]

Element	Group	%Contribution to the guideline values					
		Rice	Corn	Corn flour	Wheat flour	Corn gofio	Wheat
K	Men	2.57	6.09	3.82	5.24	7.35	6.97
	Women	3.36	7.96	5.00	6.85	9.62	9.12
Na	Men and women	-	-	0.21	1.47	-	-
Cu	Men	11.3	7.5	4.4	8.8	8.8	18.8
	Women	13.8	9.2	5.4	10.8	10.8	23.1
Mo	Men and women	76.9	61.5	30.8	61.5	92.3	123
Mn	Men and women	24.0	16.7	8.7	28.0	27.0	61.7

The consumption of 100 g/day of wheat means a contribution of 123% of the DRV of Mo set at 65 µg/day for adults [13]. Similarly, 100 g/day of wheat provides a noteworthy percentage (61.7%) of the DRV of Mn set at 3 mg/day for adults [13].

Iron deficiency in the Cape Verdean population is still considered a public health problem [40], and 20.6% of women of reproductive age (15 to 49 years old) have been diagnosed with anemia. The prevalence of anemia in non-pregnant women and pregnant women is 21% and 22.5%, respectively. According to the results of the present study, 100 g/day of wheat flour provides (bearing in mind the 15% bioavailability rate for this element [17]) a contribution percentage to the recommended Fe intake of 40.4% for men, 18.8% for premenopausal women, and 46.1% for menopausal women. However, it should be noted the non-heme form of the Fe found in cereals hinders its absorption [41]. Therefore, a lower percentage of bioavailability should be considered (5–10%), which means that the contribution percentages from the consumption of wheat flour (100 g/day) would be around 13.4–26.9% for men, 6.3–12.5% for premenopausal women, and 16.3–32.6% for menopausal women. These contribution percentages are equally noteworthy.

Considering that the Cape Verde National Food and Nutrition Plan (2021–2025) aims to reduce Fe anemia by encouraging the consumption of bio-fortified foods and by introducing means to ensure that the private sector fully complies with all the legislation regarding fortifying the iron and folic acid content of wheat flour [3], the consumption of cereals and cereal derivatives could help reduce the prevalence of anemia.

The Mg contribution from the consumption of 100 g per day of corn is also noteworthy, since this provides 36.6% (men) and 31.0% (pre- and postmenopausal women) of the Mg DRV set by the FAO (260 mg/day for men and 220 mg/day for pre- and post-menopausal women) [17]. It should be noted that unprocessed cereals or derivatives such as gofio are the most recommended products for consumption to meet the daily Mg requirement.

## Conclusions

This research work demonstrates the importance of the consumption of cereals and derivatives in the daily intake of essential elements. While wheat has high levels of Mg, Mn, and Zn, wheat flour is a relevant source of Na, Ca, and Fe for the Cape Verde population. Corn gofio is noteworthy for its high K content. The differences observed in the analyzed samples show the importance of consuming a variety of cereals as opposed to the consumption of a single cereal/derivative. The correlation study reveals negative correlations between Mo-Na and Na-Zn. This finding could suggest interference between these elements. The consumption of 100 g/day of wheat provides a notable contribution of Mo, Mn, Fe, and Mg. In addition, the fortification of cereal derivatives can counteract the decrease in the content of some essential elements in the final product. Considering the interest of the Cape Verdean public health authorities in the optimal nutritional status of its population, it is advisable to strengthen the promotion of the consumption of cereals and derivatives, especially in young populations.

## Data Availability

The data supporting the findings of the study are available in the present manuscript.
